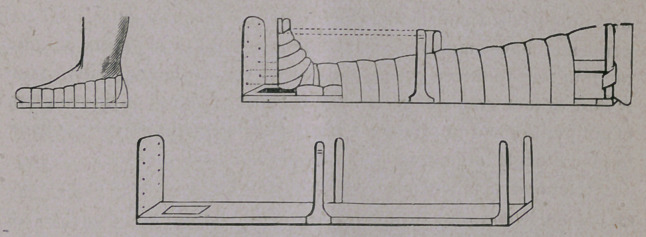# From Dr. H. Mynter’s Surgical Clinic at the General Hospital

**Published:** 1887-04

**Authors:** A. R. Davidson


### Clinical Reports.

### *FROM DR. H. MYNTER'S SURGICAL CLINIC AT THE GENERAL HOSPITAL.*

[Reported for the JOURNAL by A. R. DAVIDSON, M. D.]

GENTLEMEN—A week ago to-day you saw this patient, who, as you will remember, suffered from very large syphilitic ulcerations of left femur and crus. By aid of iodide of potash in large doses and thermocautery locally, I had succeeded in changing the old indolent ulcers, so that a healthy, granulating, but very large, ulcer was then present. I, therefore, tried to cover it with flaps of skin, after the method of Thiersch, and, as you will see, the flaps have adhered where they were transplanted. As this is a new method, I will recapitulate the leading points. While by Reverdin's method small pieces of skin were laid on the granulating surface, the method of Thiersch is different, as here we have to remove the granulations with a sharp spoon down to the firmer underlying tissues. The copious bleeding is stopped by pressure with compresses for about ten minutes, the compresses being soaked in a solution of chloride of sodium (6-10 per cent.)—60 centigrams to 100 grams. Corrosive sublimate or other strong disinfectants must not be used, as they destroy the vitality of the cells. When the bleeding is stopped, the flaps are cut out from the well-cleaned leg or arm with a razor. The flaps should be five to ten centimeters long, one to two centimeters broad, and are transferred from the razor directly on the shining surface, and by aid of two probes completely unfolded. They should be as thin as microscopical preparations. With a soft sponge, dipped in the same solution, they are pressed against the wound, and care must be taken that no air-bubbles or blood are retained under them, and that the margins lie smoothly; otherwise, pus and secretion may work in under the flaps and loosen them. After the wound is closed with the flaps, it is covered with protective, dipped in the same solution, an antiseptic occlusive bandage is applied and left undisturbed for eight days, and when then removed, you will find the flaps adherent and the wound almost healed. I told you that these flaps adhere to almost all tissues, even to bone deprived of its periost, and that we here have a method by aid of which even very large wounds may be healed with astonishing rapidity. In course of time, the flaps grow in thickness and become movable on the underlying tissues.

The next patient which I show you here has an ulceration, which I will defy any surgeon to heal by any other method than Thiersch's transplantation; yes, for which most surgeons would advise amputation. As you see, he has lost almost all the skin on the left crus, from the knee to the ankle, on account of a phlegmonous erysipelas with gangrene of all the subcutaneous tissue, leaving the fascia and all the muscles exposed. I simply show him to you now that you may get an idea of lesions, which may be treated successfully by this method.

Formation of pus may occur (1) on the surface of wounds healing by granulation; (2) on serous and mucous surfaces, as in empyema, arthroitis, peritonitis, etc.; (3) as a collection imbedded in the tissues, and bounded by an abscess membrane, an abscess, and (4) as in this case, as an infiltration in the connective tissue, with a tendency to spread, and no disposition to self-limitation. It has no abscess membrane as the abscess has, and is called purulent infiltration, diffuse inflammation, phlegmonous erysipelas, and phlegmone diffusa.

English writers apply the name phlegmonous erysipelas to the diffuse inflammations and suppurations attending erysipelas when it attacks the deeper layers of the skin and the subcutaneous tissues, while the French make a distinction between erysipelas and this disease, calling it phlegmone diffusa. Modern science has shown that they are identical, having for their cause a peculiar poison, analogous to that which Koch showed produced gangrene in mice. The effect of this poison is to produce rapid death of the tissues when brought in contact with it through the lymphatics. The poison, if it be called so, is the micro-organisms called bacteriæ, which are found everywhere where putrefaction takes place. Normal urine, for instance, is innocent if brought in contact with the subcutaneous tissues through a wound in the bladder or urethra, but putrid urine produces immediately purulent infiltration. The diffuse swelling of the fore-arm following mechanical injuries of a finger, belongs to the same class; so does inflammation from rattlesnake poison.

Purulent infiltration is characterized and distinguished from erysipelas by a more boggy feeling, deep soreness, rarely fluctuation, tendency to surface gangrene by cutting off the vascular supply and gangrene of the subcutaneous tissue. By incision, a thin, fœtid pus escapes, mixed with shreds of connective tissue; emphysema is often present, and the patient offers the usual picture of severe septicæmia. In regard to treatment, always make large incisions, as here was done, and cut as much of the sloughing tissue away with scissors as you can. Continuous water-bath is then often of great service. Roborantia and stimulating treatment becomes necessary early in the disease, if you do not succeed in arresting the progress.

The third patient has something the matter with the ankle-joint, but what this something is I doubt if any of you could tell me from the history you just have heard. The history of a patient ought to be so written that you all could form an intelligent opinion of the disease from hearing it, and then complete it by your objective examination. As this history is sorely defective in this regard, let us examine the patient together and see if we can find out what ails him. As you see, there is considerable swelling around his right ankle. Might this be of syphilitic origin? He denies ever to have had syphilis, and, as you know, syphilis is rare in the articulations, while common in bone and periosteum; attacks especially tibia, clavicle, ulna, sternum, cranial bones, nasal bones, palate and maxilla. Syphilitic lesions of bone commence late in the disease, originate mainly from gummata, and may occur as an osteoperiostitis, which appears as an infiltration of cells in the deep layers of the periosteum, while, at the same time, the Haversian canals enlarge and become filled with gelatinous material, the whole forming a soft node. If the cells undergo ossification, exostosis or hard node occurs.

Syphilis may show itself as an osteoporosis, as caries, caused by gummata, especially in the cranial bones, as necrosis, also most frequently in the cranial bones. The patient, however, has never had, nor has he now, any symptoms of syphilis.

The next question is in regard to injury. Which injuries are frequent at the ankle? We meet here especially fractures of tibia, fibula and astragalus, sprains, contusions, etc. Fractures are either direct or indirect — direct on point of injury, indirect on point of predilection. The two malleoli form a recess into which astragalus fits, so that only extension and flexion is permitted in the hinge, except when the foot is strongly extended, when some lateral movement is possible.

If a patient falls on the bottom of the foot, tibia receives the whole weight and fibula escapes. When force is applied to the inner or outer margin of the foot, malleolus externus is submitted to great pressure by abduction and adduction. In the first place, abduction, malleolus externus is forced upwards and outwards by contact with the outer surface of the os calcis, and the fibula fractures about two inches above the joint. If the violence is great, malleolus internus is fractured too, unless the ligaments are ruptured. In the second place, adduction, the malleolus externus must follow the os calcis inwards, and is then broken on a line of the joint, where the upper outer margin of the astragalus forms the pivot. The astragalus is less exposed to fractures and the cause is generally a fall on the foot. This man has received no injury which could produce fractures. Sprains and contusions may also be excluded.

He might then have an arthroitis, by which we understand an inflammation of a part or of all the constituents of a joint. An arthroitis may be acute or chronic, may come from local or constitutional causes. The local causes may be wounds, bruises, sprains, cold, etc., and the arthroitis then generally commences as a synovitis.

The constitutional causes may be rheumatism, gout, pyemia and exanthematic diseases, and, the most frequent of all, tuberculosis. If the cause be rheumatism or tuberculosis, the bones, ligaments and cartilages suffer first; if pyemia, scarlatina, etc., it commences as a purulent synovitis. The gonorrhæal arthroitis I consider of pyemic nature.

An arthroitis in the ankle-joint commences often in an insidious manner, as in this case, with soreness and stiffness, which subsides again, but soon returns, and soon is attended with swelling in those parts of the joint where the least resistance is encountered: in front and between the malleoli, later on both sides of the tendon Achilles. If the ligaments participate, lateral mobility occurs; if the cartilages and bones are inflamed, grating in the joint takes place.

Abscesses may form, except when fungous granulations are present; the swelling is then semi-elastic, the color of the skin natural. If it begins as an osteitis or epiphysitis, we find enlargement and expansion of the bones.

The pain is deep-seated, boring and gnawing, and produced by movements in the affected joint. You remember that Choparts' joint allows only rotation of the anterior part of the foot, the astragalo-calcaneus articulation abduction and adduction, the astragalo-crural articulation extension and flexion. If we now return to our patient, then he tells us that he comes from healthy parentage; has never been sick before. His disease commenced some two months ago without known cause, with pain, stiffness and swelling around right ankle-joint, which subsided again and then returned. He has been unable to use it since. If you look at the ankle, you discover a diffuse swelling of the whole joint, it being two inches more in circumference than the left. The swelling is most prominent in front between the malleoli and behind on both sides of the tendon Achilles, the skin has its normal color, the feeling is semi-elastic and there is no pus. I punctured it yesterday with a hypodermic syringe to satisfy myself on that point. The pain is excruciating by flexion and extension in the astragalo-crural articulation, and is much relieved by extension in this joint. No pain by abduction and adduction or rotation.

We find, besides, slight lateral mobility. You see, therefore, that all the symptoms point to a subacute or chronic affection of this joint, and, I believe, we safely can say that he has an osteo-arthroitis of the ankle-joint, probably of tuberculous nature We must not forget that inflammation of tendons and caries of astragalus may produce similar symptoms; but in inflammations of tendons, the swelling appears first behind and following the course of the tendons; in caries astragali, the swelling appears some distance anteriorly to the astragalo-crural joint. What is the prognosis of our patient? We can agree upon one point: that he has a serious lesion, which in time, and perhaps very soon, may necessitate serious operations, probably re-section of the joint.

How shall we then treat him? It would be good surgery already now to open the joint, and then act according to what we find — drain, if the bones are healthy; resect, if they are diseased; extirpate the capsule, if tuberculous, etc. But the great relief he finds by extension of the joint, and his good bodily condition, makes me inclined to wait some time and try methodical extension and elastic pressure. Ignipuncture has been used frequently, recommended especially by Dr. Roswell Park, and I should use that if I knew that the trouble had started as an epiphysitis, and if there were reasons to believe that it yet was local. To go into a joint, we do not know where or why, with a red-hot iron, I scarcely consider advisable.

It has always been rather difficult to apply extension to the ankle-joint, but I will show you here an apparatus, invented by my friend, Dr. Hansmann, first assistant surgeon in the General Hospital in Hamburg, which I consider excellent. It consists of a splint about eighty centimeters long, with an upright foot-piece and two upright bars on each side. The foot, when lying on the splint, is about twenty centimeters from the foot-piece. A wooden sole is put under planta pedis, and fastened with strips of adhesive plaster (Fig. 1), and three screws are then inserted on each side of the wooden sole, to fasten the strings to. The foot-piece and the lower bar are perforated with holes, through which screws pass, terminating in hooks. The two upper bars are used to fasten two long pieces of adhesive plaster, by aid of which the contra-extension is made from the femur. The apparatus may be used with advantage in pedes vari and valgi, fractures of the ankle-joint, inflammations in the astragalo-crural joint, for after-treatment after Phelps' operation, etc. We will apply it here, and wait a time to see the result.

## Figures and Tables

**Figure f1:**